# Aspirin for the primary prevention of skin cancer: A meta-analysis

**DOI:** 10.3892/ol.2015.2853

**Published:** 2015-01-07

**Authors:** YUN ZHU, YANG CHENG, RONG-CHENG LUO, AI-MIN LI

**Affiliations:** 1 Cancer Center, Southern Medical University, Guangzhou, Guangdong 510515, P.R. China; 2 Digestive Department of Nanfang Hospital, Southern Medical University, Guangzhou, Guangdong 510515, P.R. China; 3 Hospital of Integrated Traditional Chinese and Western Medicine, Southern Medical University, Guangzhou, Guangdong 510315, P.R. China

**Keywords:** skin cancer, aspirin, primary prevention, meta-analysis

## Abstract

Skin cancer is one of the most common cancers worldwide. There are three major skin cancer types: basal cell carcinoma, squamous cell carcinoma and malignant melanoma. General risk factors for skin cancer include fair skin, a history of tanning and sunburn, family history of skin cancer, exposure to ultraviolet rays and a large number of moles. The incidence of skin cancer has increased in the USA in recent years. Aspirin intake is associated with chemoprotection against the development of a number of types of cancer. However, whether aspirin intake can reduce the risk of development of skin cancer is unclear. The present meta-analysis of available human studies is aimed at evaluating the association between aspirin exposure and the risk of skin cancer. All available human observational studies on aspirin intake for the primary prevention of skin cancer were identified by searching MEDLINE (Pubmed), BIOSIS, EMBASE, Cochrane Library and China National Knowledge Infrastructure prior to March 2013. The heterogeneity and publication bias of all studies were evaluated using Cochran’s Q and I^2^ statistics, followed by a random-effect model where applicable. The pooled data were analyzed by odds ratios (ORs) and 95% confidence intervals (CIs). A total of eight case-control and five prospective cohort studies from 11 publications were selected for this analysis. There was no evidence of publication bias in these studies. Statistical analyses of the pooled data demonstrated that that a daily dose of 50–400 mg aspirin was significantly associated with a reduced risk of skin cancers (OR, 0.94; 95% CI, 0.90–0.99; P=0.02). Stratification analysis indicated that the continual intake of low dose aspirin (≤150 mg) reduced the risk of developing skin cancer (OR, 0.95; CI, 0.90–0.99; P=0.15) and that aspirin intake was significantly associated with a reduced risk of non-melanoma skin cancers (OR, 0.97; CI, 0.95–0.99; P=0.22). Overall, these findings indicated that aspirin intake was associated with a reduced risk of developing skin cancer. However, more well-designed randomized controlled trials to measure the effects of aspirin intake are required to confirm this.

## Introduction

Skin cancer is one of the most common malignancies in the USA, where >2,000,000 cases are diagnosed annually (1). There are several types of skin cancers, including basal cell carcinoma (BCC), squamous cell carcinoma (SCC), and malignant melanoma (MM). BCC and SCC are collectively termed non-melanoma skin cancer (NMSC). BCC usually presents as a painless raised area of skin with an ulcer, which may damage surrounding tissues, however, it is unlikley to metastasize to distant organs. SCC may also form an ulcer, and often presents as a hard red lump with a flat scaly surface. SCC is more likely to metastasize to distant organs. Melanomas are the most aggressive type of skin cancer, which present as a large, uneven mole that has changed in color (2). The incidence of skin cancer, particularly MM, is increasing, with an annual growth rate of 3–5% in the USA, and appropriate preventive approaches are urgently required (3,4). Currently, known risk factors for the development of skin cancers include fair skin, blue or green eyes, blond or red hair, multiple moles, excess ultraviolet (UV) radiation from sun exposure, and a history of severe sunburn and skin cancer (5). Primary strategies to prevent the development and occurrence of skin cancers include reducing skin cancer-related risk behaviors by avoidance of UV over-exposure, and by the regular use of sunscreen creams (6,7).

Inflammation is associated with the development of malignant tumors, particularly for epithelial cell tumors, including skin cancers (8,9). Cyclooxygenase-2 (COX-2) controls prostaglandin synthesis, regulating inflammation and the development and progression of malignant tumors. Furthermore, COX-2 can positively regulate antiapoptotic, proangiogenic and other tumorigenic processes, and is upregulated in human skin cancers (10,11). Accordingly, chemotherapies with COX-2 inhibitors and non-steroidal anti-inflammatory drugs (NSAIDs) have been tested for the prevention of tumors in humans (12). Epidemiologically, treatment with aspirin/NSAIDs benefits patients with various solid cancers, such as colon cancer, esophageal cancer, and breast cancer (13). However, a previous meta-analysis revealed no significant protective effect of non-aspirin NSAIDs in preventing the development of skin cancers in humans (14).

Aspirin, also known as acetylsalicylic acid, is a salicylate drug with analgesic, antipyretic and anti-inflammatory activity. Aspirin is an inhibitor of COX-1 and COX-2, predominantly affecting COX-1 (15). The efficacy of treatment with aspirin for the prevention of tumor development remains controversial. While several epidemiological studies have demonstrated that treatment with aspirin may reduce the incidence of skin cancers, other studies have yielded conflicting results (16–26). Therefore, in the present study, a meta-analysis was performed to evaluate the effect of aspirin on the primary chemoprevention of skin cancer according to the available clinical observational studies.

## Materials and methods

### Search strategy

A systematic literature search of MEDLINE (Pubmed), BIOSIS, EMBASE, Cochrane Library, and China National Knowledge Infrastructure was conducted to identify cohort and case-control studies on aspirin intake and skin cancer development, published between January 1980 and March 2013. The following medical subject headings or keywords were used, without language restriction: i) ‘Aspirin’, ‘non-steroidal anti-inflammatory drugs’ or ‘acetylsalicylic acid’; ii) ‘skin cancer’, ‘skin tumor’, ‘melanoma skin cancer’, ‘non-melanoma skin cancer’, ‘squamous cell carcinoma’, or ‘basal cell carcinoma’. The cited references in retrieved articles were also screened to identify any additional relevant studies.

### Study selection

The titles and abstracts of individual publications were screened, and the nature of each study was evaluated independently by two reviewers (Zhu and Cheng). The studies were included if they met all of the following criteria: i) Had a case-control or cohort design, ii) evaluated exposure to aspirin, iii) reported occurrence of skin cancer diagnosis, and iv) reported the adjusted relative risks (RRs), hazard ratios (HRs), or odds ratios (ORs), as well as the corresponding 95% CI. If publications were duplicated or if articles came from the same study population, the study with the largest sample size was included.

### Data extraction and quality assessment

Data were extracted from individual publications by two reviewers (Zhu and Cheng), independently and in a blinded manner (without prior knowledge of the year of publication, author and journal). The extracted data included authors, publication year, population, sample size, medication type and frequency of use, information source for measurement of aspirin exposure and for identification of skin cancer cases (e.g. questionnaire, interview, pharmacy database), ORs or RRs with and without adjustment for potential confounders, potential confounders used for adjustment (e.g. age, skin color) and the study design (cohort vs. case-control). If there was a disagreement, the data were further discussed by two reviewers until a consensus was reached.

The methodological quality of each study was assessed using the Newcastle-Ottawa Scale (NOS) (27), by evaluation of the following three areas: The selection of study groups, comparability of groups, and ascertainment of either the exposure or outcome of interest for case-control or cohort studies, respectively. If a study had a score ≥5 out of a maximum score of 9, the study was considered to be of high-quality.

### Data analysis and risk of bias

All aspirin-related chemopreventive studies of skin cancer were analyzed simultaneously and further stratified, according to study design (cohort vs. case-control), method for determining exposure to aspirin (i.e. questionnaire vs. pharmacy database), method for identifying skin cancer cases (self-reported vs. medical records and pathology), histological type (SCC vs. BCC vs. MM), gender (men vs. women), duration of medication use, and study population (American vs. European).

Potential publication bias was assessed using qualitative and quantitative methods. Initially, it was evaluated by funnel plots of the ORs versus their standard errors, and subsequently by the Begg’s test (rank correlation method) (28) and Egger’s test (linear regression method) (29). P>0.10 was considered to indicate no publication bias.

### Statistical analysis

OR was used as a common measure across all studies for determining the degree of a potential association between aspirin intake and risk of development of skin cancer. The RRs and HRs were directly considered as ORs. The potential heterogeneity in the results across the studies was examined by Cochran’s Q and I^2^ statistics (30). If a P value for heterogeneity was <0.10 or I^2^ was >50%, substantial heterogeneity was considered and the summary was estimated on the basis of the random-effect model, as described by DerSimonian and Laird (31). The sensitivity was analyzed by excluding each study individually to evaluate the consistency of our results. All analyses were performed using STATA version 10.0 (StataCorp LP, College Station, TX, USA).

## Results

### Literature search

**Figure 1:**
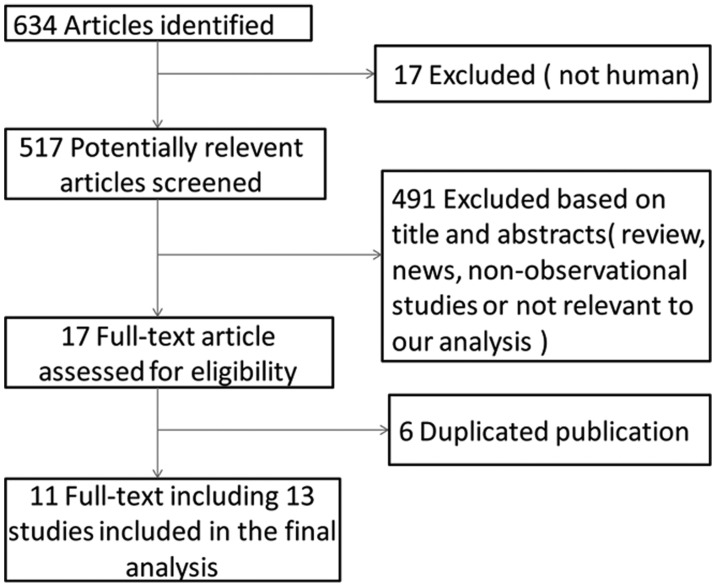
Flow chart illustrating the literature search for studies on aspirin intake and a risk of skin cancer.

A total of 634 relevant publications were identified by a systematic literature search, and 623 out of 634 publications were excluded due to duplications or various other reasons (e.g. if the publications were review papers or news articles, or related to randomized controlled studies or animal experiments), according to the titles and abstracts. Finally, eight case-control studies (16,19,22,24–26) and five cohort studies (17,18,20,21,23) were included in the meta-analysis. A flow chart (Fig. 1) illustrates the process of selection of relevant studies.

### Study characteristics and quality assessment

According to the inclusion criteria, a total of eight case-control studies with 21,356 cases and 187,037 controls and five cohort study incorporating 294,377 participants were included in the meta-analysis. The main characteristics of these studies are summarized in Table I. All research literature was in English; three studies were based in Denmark (24), one in the Netherlands (25), and nine in the USA (16–23). Seven studies (16–19,21,22,24–26) included both genders and two studies included only females (20,23). The majority of studies had matched cases and controls, and had adjusted for a wide range of potential confounders, including age, gender, ethnicity, skin color, hair color, amount of sun exposure, history of severe sunburns, number of moles, family history of skin cancer, smoking status and other factors.

The quality scores of these studies are summarized in Table IIA and IIB. The range of quality scores was from 6–9. The average scores of case-control studies and cohort studies were 7.8 and 8.0, respectively. All studies were considered to be of high-quality.

### Overall analyses and bias assessment

**Figure 2:**
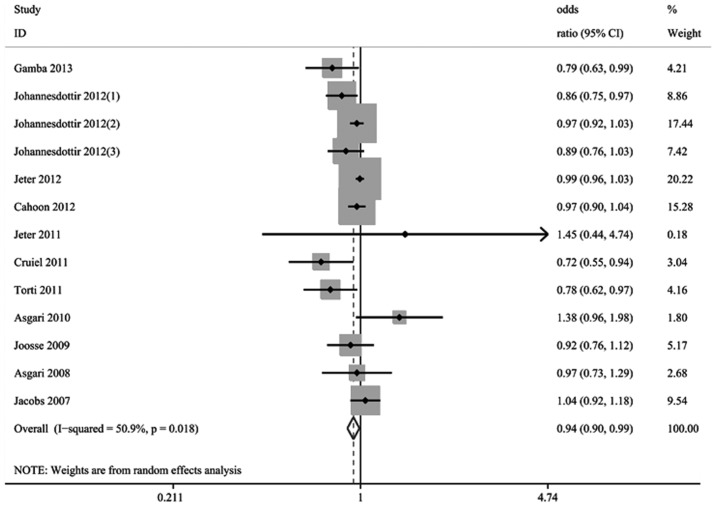
Forest plot showing the association between aspirin intake and reduced risk of skin cancer. CI, confidence interval.

**Figure 3:**
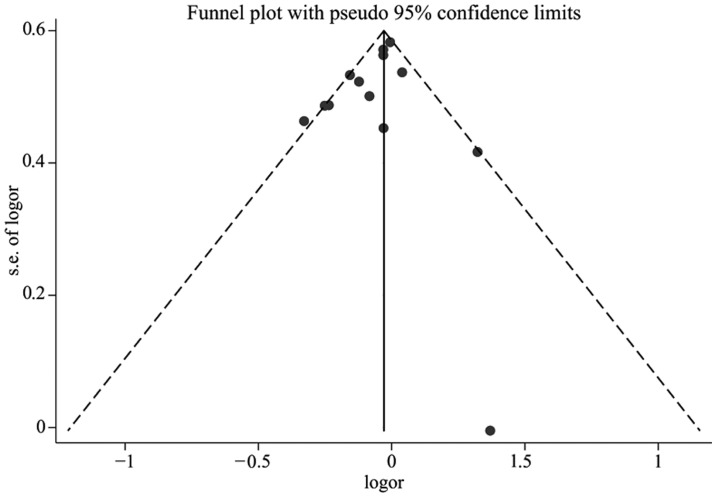
Funnel plot of studies on aspirin intake and risk of skin cancer.

All studies reported OR and 95% CI for aspirin exposure and risk of skin cancer after adjusting for confounding factors. The pooled results indicated that regular aspirin exposure decreased the risk of developing skin cancers by 6% (OR, 0.94; 95% CI, 0.90–0.99). Statistical analyses revealed significant heterogeneity among the studies (P=0.02; I^2^=50.9%; Fig. 2). The sensitivity analyses, by excluding any single study in each step, revealed that only one [Jeter *et al* (23)] out of 13 studies included was considered to have a high risk of differential-verification bias. Exclusion of this study decreased the heterogeneity, but did not alter the results (OR, 0.95; 95% CI, 0.91–0.98; P=0.04; I^2^= 47.1%). No indication of a publication bias was identified either from the funnel plot (Fig. 3), or from the Egger’s test (P=0.17) or Begg’s test (P=0.67).

### Subgroup analysis

The effects of aspirin intake on the risk of skin cancer in subgroup meta-analyses are shown in Table III. Compared with the overall analysis, the results from individual subgroup analyses were similar: Case-control studies (OR, 0.90; 95% CI, 0.82–0.99; P=0.03; I^2^=53.9%), medical record of skin cancer (OR, 0.95; 95% CI, 0.92–0.99; P=0.08; I^2^=40.1%), and continual intake of low dose aspirin (OR, 0.95; 95% CI, 0.90–0.99; P=0.15; I^2^=40.0%). Aspirin intake exerted significant protective effects against the development of SCC (OR, 0.90; 95% CI, 0.82–0.98; P=0.22; I^2^=31.7%) and in the non-American population (OR, 0.94; 95% CI, 0.90–0.99; P=0.29; I^2^=20.7%), whilst it had marginal protective effects on the development of BCC (OR, 0.98; 95% CI, 0.95–1.00; P=0.64; I^2^=0%). However, no significant protective effects were observed in the other relevant strata.

### Sensitivity analysis

In the sensitivity analyses, the combined results were recalculated by excluding one study per iteration. After excluding one particular study [Jeter *et al* (23)], the remaining studies retained significant heterogeneity, and indicated that aspirin exposure had significant protective effects on MM. However, exclusion of the Jeter *et al* (23) study reduced the heterogeneity among the remaining studies and indicated that short term aspirin intake may decrease the risk of skin cancer in females (data not shown).

## Discussion

The results of the current study extend and support the previous observation that aspirin intake is associated with a decreased risk of developing skin cancer. However, the results must be interpreted with caution, due to the substantial heterogeneity among the studies included in this meta-analysis. This was anticipated given the difference in the study populations, study designs, gender and age of the participants, the method of ascertainment of patients and dosage and duration of medication, follow-up time and adjustment variables across studies. The sensitivity analyses indicated that the study conducted by Jeter *et al* (23) potentially caused significant heterogeneity in the pooled data, as this study was conducted in well-educated nurses with greater awareness of health concerns.

The current study included high-quality observational studies on aspirin intake for the primary prevention of skin cancer. The results from the case-control studies indicated a significant protective association between aspirin intake and a reduced risk of primary skin cancers, while the results from the cohort studies indicated only a borderline significance in the protective effects of aspirin intake against skin cancer.

Cohort studies are regarded to be the most accurate observational studies, however, the value of a cohort study depends on its overall quality. The report by Jeter *et al* (23) only comprised nurses, whilst Jacobs *et al* (21) and Asgari *et al* (15) did not confirm skin reactions to sun exposure, family history, and the number of moles, which are the main risk factors for skin cancers. Studies have reported a marked increase in the incidence among the younger population, particularly in females <40 years of age, which increased from 28.8 individuals per 100,000 of the population in 1990 to 33.1 individuals per 100,000 of the population in 2000 (32). However, Gamba *et al* (20) studied postmenopausal females only and Asgari *et al* (17) studied subjects aged between 50 and 76 years, therefore these two cohort studies were not representative of the whole population. The absence of validated reports of aspirin use with prescription records should also be taken into account when interpreting these results.

The results from case-control studies in general must be interpreted with caution due to the methodological limitations. Jeter *et al* (22) studied the spouses of patients as the controls, and this may cause have potential selection bias in the control group as a number of spouses of the studied subjects did not participate in the study. Johannesdottir *et al* (24), studied cases identified through the Danish Cancer Registry, in which only ~60% of SCC and BCC cases were recorded. The incompleteness of tumor records may also cause a potential bias affecting the results. In addition, a number of the case-control studies had a moderate sample size, which may overestimate the treatment effect.

Stratification analyses indicated that aspirin intake reduced the risk of development of NMSC, but not of MM. Similarly, aspirin intake had a more significant protective effect against the development of SCC than BCC. This difference may be attributed to the differential levels of COX expression in these different types of skin cancers. Indeed, COX-2 expression is upregulated in SCC, whilst levels of COX expression in BCC and MM are controversial (33–35).

In addition, we observed that aspirin intake exerted borderline statistically significant effects on the development of skin cancers between females (OR, 0.88; 95% CI, 0.74–1.04) and males (OR, 0.85; 95% CI, 0.68–1.07). Gamba *et al* (20) demonstrated that aspirin intake may be chemopreventive against the development of melanoma in postmenopausal women, consistent with a previous study that indicated a similar effect against colorectal cancer (36). However, two studies on aspirin intake for preventing breast cancer obtained conflicting results in postmenopausal females (37,38). Further studies into the potential association of aspirin intake with protection from skin cancer in postmenopausal females are required to gain further insight.

Stratification analyses also revealed that low dose aspirin intake (≤150 mg) exerted a marginal protective effect on the development of skin cancer, while high dose aspirin intake (>150 mg) did not show any protective effect. However, the categories of aspirin dosages varied across the studies, and the estimated dose in individual studies was based on study-specific definitions; 150 mg was set as a cut off value. A daily dose of ≤150 mg was considerd to be a ‘low-dose’ while a daily dose of >150 mg was considerd to be a ‘high-dose’. Thus, the effect of aspirin intake may be better considered as an inverse dose-risk correlation.

Additionally, stratification analyses indicated that aspirin intake for a short (≤5 years) or long (>5 years) time period was associated with a reduced risk of development of skin cancers. These results may stem from limited sample sizes in some groups of subjects, leading to less power to achieve a meaningful conclusion. Continual intake of aspirin has, however, been associated with a reduced risk of other types of tumors (39).

Aspirin intake had a marginal protective effect against the development of skin cancer in Americans, however, the study also revealed a significant protective effect against skin cancer in other Caucasian populations. The varying association levels may be due to the dissimilarities in the baseline risk of skin cancer between these populations.

There were several potential limitations to the present meta-analysis. Firstly, the analysis was based solely on observational studies, which identify only the potential association between the two factors, and not causality. Secondly, considerable heterogeneity was present among the included trials, which may have impacted the results. Thirdly, some patients taking aspirin may also have taken other NSAIDs, which may confound the results, yet few studies have adjusted for this factor. Further large-scale, well-designed randomized controlled trials are needed to validate the protective effect of aspirin intake on the development of skin cancer.

In summary, the current meta-analysis of observational studies indicated that aspirin intake, particularly with continual small doses, was significantly associated with a reduced risk for the development of skin cancer, primarily SCC and BCC, in both females and males. These findings may have important public health implications. However, the causative protection against skin cancers by aspirin intake remains to be confirmed.

## Figures and Tables

**Table I:** Characteristics of epidemiological studies of aspirin intake and skin cancer risk included in the meta-analysis.

Study	Ethnicity	Design	Gender	Cancer type	Participants, n	Patients, n	Exposure source	Cancer confirmed	Confounders included in adjusted estimates
Gamba 2013 (20)	USA	Cohort	Female only	MM	59,806	548	Prescription records	Medical records	a1,a2,b,c1,d1,e1,h1,l,m,p1,r1,s1,s2,s3,t1
Jeter 2012 (23)	USA	Cohort	Female only	MM	92,125	658	Self-reported	Self-reported	a1,a2,b,f1,h2,n,p1,p2,
				SCC		1,337		Self-reported	q,r1,s1,s3,s4,v1,v2
				BCC		15,079		Medical records	
Johannesdottir 2012[1] (24)	Denmark	Case-control	Female and male	MM	196,529	3,089	Prescription records	Pathology	a1,c2,d2,g1,g2,t2
Johannesdottir 2012[2] (24)	Denmark	Case-control	Female and male	SCC		1,921	Prescription records	Pathology	a1,c2,d2,g1,g2,t2
Johannesdottir 2012[3] (24)	Denmark	Case-control	Female and male	BCC		12,864	Prescription records	Pathology	a1,c2,d2,g1,g2,t2
Cahoon, 2012 (18)	USA	Cohort	Female and male	BCC	58,213	2,291	Self-reported	Medical records and self-reported	a1,a2,g1,r1
Jeter 2011 (22)	USA	Case-control	Female and male	MM	446	327	Self-reported	Medical records	a1,f1,g1,n,s5
Cruiel 2011 (19)	USA	Case-control	Female and male	MM	1,000	400	Self-reported	Self-reported	a1,g1, s4,t2
Torti 2011 (26)	USA	Case-control	Female and male	SCC	1,484	535	Self-reported	Medical records	a1,c1,g1,s3,s4,s5
				BCC		487			
Asgari 2010 (16)	USA	Case-control	Female and male	SCC	830	415	Self-reported and prescription records	Medical records	h2, o
Joosse 2009 (25)	Netherlands	Case-control	Female and male	MM	8,104	1,318	Prescription records	Medical records	a1,d3,g1,g2,s7,t2,y
Asgari 2008 (17)	USA	Cohort	Female and male	MM	63,809	349	Self-reported	Medical records	a1,c3,d3,e1,e2,f1, f2,g1,h1,h3,s4,v3
Jacobs 2007 (21)	USA	Cohort	Female and male	MM	18,127	1,049	Self-reported	Medical records	a1,b,d3,e1,g1, g2,m,r2,p1,s3

a1, age; a2, acetaminophen or non-aspirin non-steroidal anti-inflammatory drugs use; b, body mass index; c1, childhood and current summer sun exposure; c2, Charlson Comorbidity Index score; c3, chronic pain in last year; d1 duration of aspirin use; d2, drugs with pigmenting adverse effects; d3, disease of cardiovascular system, kidney, ulcer or arthritis; e1, education; e2, ever had moles removed; f1, family history of skin cancer; f2, freckles between ages 10 and 20 years; g1, gender; g2, glucocorticoids or other immunosuppressive medication use; h1, history of skin cancer; h2, height; h3, hair color; l, last medical visit; m, medical indication; n, number of moles; o, occupational sun exposure; p1, physical activity; p2, postmenopausal; q, questionnaire cycle; r1, regional solar radiation; r2, race; s1, skin reaction to the sun; s2, suncreen use; s3, smoking status; s4, times of sunburns over life; s5, skin color; t1, times since last medical visit; t2, town matched; v1, Vitamin D intake; v2, Vitamin C intake; V3, multivitamin intake; y, Duration of disease (years). MM, malignant melanoma; SCC, squamous cell carcinoma; BCC, basal cell carcinoma.

**Table II:** 

A, Methodological quality of case-control studies									

	Selection^a^					Exposure^a^			
			
Study	Adequate definition of cases	Representativeness of cases	Selection of control subjects	Definition of control subjects	Comparability^b^	Exposure assessment	Same method of ascertainment for all subjects	Non-response rate	Total scores
Johannesdottir 2012[1] (24)	*	*	*	*	**	*	*	-	8
Johannesdottir 2012[2] (24)	*	*	*	*	**	*	*	-	8
Johannesdottir 2012[3] (24)	*	*	*	*	**	*	*	-	8
Jeter 2011 (22)	*	*	-	*	*	*	*	-	6
Cruiel 2011 (19)	*	*	*	*	**	*	*	-	8
Torti 2011 (26)	*	*	*	*	**	*	*	-	8
Asgari 2010 (16)	*	*	*	*	**	*	*	-	8
Joosse 2009 (25)	*	*	*	*	**	*	*	-	8

B, Methodological quality of cohort studies									

	Selection^a^					Outcome^a^			
			
Study	Representativeness of exposed cohort	Representativeness of nonexposed cohort	Ascertainment of exposure	Outcome of interest	Comparability^b^	Assessment of outcome	Length of follow-up^c^	Adequacy of follow-up^d^	Total scores

Gamba 2013 (20)	*	*	*	*	**	*	*	*	9
Jeter 2012 (23)	-	*	*	*	*	*	*	*	7
Cahoon 2012 (18)	*	*	*	*	**	*	-	*	8
Asgari 2008 (17)	*	*	*	*	**	*	-	*	8
Jacobs 2007 (21)	*	-	*	*	**	*	*	*	8

a A study could be awarded a maximum of one star for each item.

b A maximum of two stars could be awarded for this item. Studies that controlled for age received one star, whereas studies that controlled for other important confounders, such as gender, received an additional star.

c A cohort study with a median follow-up time of >8 years was assigned one star.

d A cohort study with a follow-up rate of >75% was assigned one star.

**Table III:** Summary odds ratios of the association between aspirin intake and skin cancer risk.

	OR^a^	95% CI^a^	I^2^, %	P-value for homogeneity	Studies, n
Study design					
Case-control	0.90	0.82–0.99	53.9	0.03	8
Cohort	0.99	0.96–1.02	20.3	0.29	5
Histological type					
NMSC	0.97	0.95–0.99	25.9	0.22	6
SCC	0.90	0.82–0.98	31.7	0.22	4
BCC	0.98	0.95–1.00	0.0	0.64	4
MM	0.96	0.82–1.12	69.3	0.00	7
Exposure determination					
Prescription records	0.92	0.84–1.01	52.6	0.06	6
Self-reported	0.98	0.96–1.01	45.6	0.07	7
Disease determination					
Medical records	0.95	0.92–0.99	40.1	0.08	11
Self-reported	0.87	0.64–1.19	81.7	0.02	2
Gender					
Female	0.88	0.74–1.04	62.1	0.05	4
Male	0.85	0.68–1.07	0.0	0.34	2
Duration of aspirin use					
Short term	0.92	0.83–1.04	66.0	0.00	8
Long term	0.90	0.78–1.05	69.1	0.00	8
Dose effects					
High dose	1.01	0.90–1.14	0.0	5.39	7
Low dose	0.95	0.90–0.99	40.3	0.15	5
Study population					
American	0.95	0.88–1.02	56.5	0.02	9
Non-American	0.94	0.90–0.99	20.7	0.29	4

a Odds ratio; all summary estimates use data adjusted for some potential confounding factors.

CI, confidence interval; NMSC, non-melanoma skin cancer; SCC, squamous cell carcinoma; BCC, basal cell carcinoma; MM, malignant melanoma.
